# Research on the Edge–Discrepancy Collaborative Method for Defect Detection in Casting DR Images

**DOI:** 10.3390/ma19050900

**Published:** 2026-02-27

**Authors:** Yangkai He, Yunxia Chen

**Affiliations:** 1 School of Intelligent Manufacturing and Control Engineering, Shanghai Polytechnic University, Shanghai 201209, China; 20241513125@sspu.edu.cn; 2 School of Materials Science and Engineering, Shanghai Jiao Tong University, Shanghai 200240, China

**Keywords:** casting DR images, defect detection, YOLOv11, MSEES, TripletAttention, SDAGFusion

## Abstract

To address the limited detection accuracy of casting defects—including pores, inclusions, and looseness—in digital radiography (DR) images, which stems from their small scale, high morphological variability, and interference from complex background textures, we propose MTS-YOLOv11: an edge–discrepancy collaborative defect detection framework tailored for casting DR imagery. Built upon YOLOv11, MTS-YOLOv11 incorporates three key innovations: (1) a Multi-Scale Edge Information Enhancement System (MSEES), integrated into the C3K2 module of the backbone network, to strengthen discriminative feature extraction for minute defects; (2) a TripletAttention mechanism embedded in high-level backbone stages to jointly calibrate channel–spatial dependencies and suppress texture-induced spurious responses under complex backgrounds; (3) a Scale-Discrepancy-Aware Gated Fusion (SDAGFusion) module positioned immediately before the detection head, enabling scale-discrepancy-aware gated fusion of multi-scale features, emphasizing defect regions while suppressing background interference. Experimental results show that on the casting DR dataset, MTS-YOLOv11 achieves mAP@0.5 = 96.5% and mAP@0.5:0.95 = 68.5%—improvements of 1.3 and 1.2 percentage points over the baseline YOLOv11—across all three defect categories. Moreover, on the same platform, MTS-YOLOv11 achieves an inference speed of 359.07 FPS, compared with 346.86 FPS for the baseline. Meanwhile, the model has 2.72M parameters and 7.8G FLOPs. These results indicate a consistent improvement in detection accuracy while maintaining a practical balance between precision and computational efficiency. Moreover, cross-dataset generalization tests on newly acquired industrial DR data show that MTS-YOLOv11 consistently outperforms the baseline across evaluation metrics, suggesting improved robustness to unseen imaging conditions and supporting its potential for real-world foundry inspection.

## 1. Introduction

With the acceleration of global industrialization, cast components—valued for their high strength, dimensional accuracy, excellent wear resistance, and relatively low production cost—have become indispensable in mission-critical sectors including aerospace, automotive manufacturing, rail transit, and heavy industrial equipment [1]. Yet, during casting, molten metal is highly susceptible to process-induced disturbances such as gas entrapment, nonmetallic inclusion, and thermally induced solidification heterogeneity, which frequently give rise to internal defects including pores and inclusions. Left undetected, these defects can precipitate catastrophic structural failures, significantly degrade mechanical integrity (e.g., fatigue life and tensile strength), compromise operational safety, and incur substantial economic losses across the product lifecycle [2]. Consequently, automated, high-accuracy detection of internal casting defects has emerged as a pivotal quality assurance step for ensuring both component reliability and system-level operational resilience.

Currently, industry-standard non-destructive testing (NDT) techniques include radiographic testing (RT) [3], ultrasonic testing (UT) [4], and magnetic particle testing (MT) [5]. Among these, X-ray-based inspection has gained prominence as a mainstream industrial modality owing to its unique capacity to penetrate geometrically complex castings, resolve sub-surface anomalies with high contrast sensitivity, and seamlessly integrate with digital image analysis pipelines. DR, a modern implementation of X-ray RT [6], delivers intuitive, high-fidelity visualization of defect type, morphology, and spatial distribution—making it one of the most widely deployed NDT methods in foundry quality control. Nevertheless, conventional DR workflows remain heavily dependent on manual interpretation by expert inspectors—a practice that is inherently time-consuming, labor-intensive, and vulnerable to inter-operator variability and cognitive bias. This subjectivity undermines detection repeatability and throughput scalability, thereby impeding the adoption of DR in high-volume, precision-driven industrial inspection environments [7].

To overcome the inherent limitations of manual DR image interpretation—including low throughput, inter-inspector inconsistency, and fatigue-induced errors—automated defect detection methods have been increasingly adopted. Early solutions predominantly relied on conventional image processing techniques. While such methods can achieve moderate accuracy in controlled settings, they are fundamentally constrained by their dependence on hand-crafted features (e.g., texture histograms, edge gradients, or morphological descriptors). Consequently, even minor variations in defect morphology, orientation, or background context necessitate labor-intensive feature engineering or parameter re-optimization, resulting in poor robustness to real-world imaging variability and severely limited generalizability across diverse casting geometries and alloy systems. Moreover, their multi-stage, rule-based pipelines lack end-to-end adaptability and are typically restricted to idealized, low-complexity inspection scenarios [8,9].

With the rapid advancement of deep learning, convolutional neural network (CNN)-based object detection frameworks [10] have emerged as a dominant paradigm in industrial vision research and deployment [11]. Unlike conventional image processing methods—which require explicit, domain-specific feature engineering—deep learning models automatically learn hierarchical, high-level semantic representations directly from raw pixel data via multi-layer nonlinear transformations. This end-to-end feature abstraction significantly enhances detection robustness across heterogeneous backgrounds, variable defect scales, and morphologically diverse casting anomalies. Early deep learning detectors were predominantly two-stage architectures, exemplified by Region-based CNN (R-CNN) [12] and its accelerated variant Faster R-CNN [13]. While achieving state-of-the-art accuracy on benchmark datasets, these models incur substantial computational overhead, exhibit slow inference latency, and lack real-time capability—rendering them impractical for high-throughput, inline industrial inspection systems with strict cycle-time constraints. To address this bottleneck, Redmon et al. introduced the pioneering one-stage detector You Only Look Once (YOLOv1) in 2016 [14], which unified detection into a single regression task and achieved near real-time throughput (≥30 FPS) with competitive accuracy—thereby establishing a viable foundation for production-grade deployment. Subsequent generations—including YOLOv5 [15,16], YOLOv7 [17,18], and YOLOv8 [19,20]—have systematically refined architectural design, optimization strategies, and loss formulations, consolidating the YOLO family as the de facto standard for industrial defect detection due to their compelling combination of end-to-end trainability, low-latency inference, precise localization, and hardware-friendly deployment efficiency. More recent iterations, such as YOLOv11 [21,22] and YOLOv12 [23], further advance this trade-off through adaptive feature pyramid designs, enhanced neck modules, and improved training schedulers, yielding superior accuracy–efficiency balance, particularly under challenging conditions including complex background textures and concurrent multi-defect occurrences. Concurrently, Transformer-based alternatives have gained traction: Real-Time Detection Transformer (RT-DETR) [24,25], an optimized derivative of DETR [26], replaces hand-crafted anchor mechanisms with global self-attention modeling and eliminates non-maximum suppression (NMS), enabling end-to-end detection with both higher accuracy and markedly reduced inference latency—especially advantageous for large-scale defects and geometrically intricate casting structures. In parallel, a growing body of work focuses on targeted architectural adaptation rather than wholesale model replacement. For instance, Andriosopoulou et al. [27] fused Faster R-CNN’s region proposal strength with YOLOv5’s speed via ensemble transfer learning to achieve accurate, real-time surface defect detection in high-pressure die-casting. Cheng et al. [28] targeted internal weld defect detection and improved the YOLOv5s architecture by replacing the original convolution modules and introducing DIoU and CIoU loss functions, which effectively enhanced both detection accuracy and speed while reducing model parameters to enable real-time efficient detection. Wu et al. [29] addressed the issues of low small-target detection accuracy and large parameter count of YOLOv7-Tiny on aluminum alloy weld DR images by introducing SPD-Conv layers and the SimAM attention mechanism to strengthen shallow feature representation, thereby significantly improving small-defect recognition performance. Chu et al. [30] further improved the YOLOv8 algorithm by integrating dilated convolutions to enlarge the receptive field, combining the SimAM attention mechanism to enhance feature focusing, and adding an additional small-object detection head, ultimately achieving high-accuracy and high-efficiency detection of casting defects.

Although existing object detection algorithms have achieved certain success in industrial defect inspection, they still face substantial challenges when detecting casting defects—such as pores, inclusions, and looseness—in DR images. These defects are typically small in size, highly variable in morphology, and low in contrast against the background, causing high-frequency edge/texture details of small targets to attenuate easily during feature extraction and downsampling. As a result, tiny-defect features are difficult to capture effectively, and because the grayscale values of defect regions are often close to those of the surrounding matrix, localization accuracy degrades. Meanwhile, under complex metallurgical textures and multi-defect co-occurrence, insufficient modeling of channel–spatial dependencies can lead to false positives and localization drift. In addition, feature fusion often suffers from scale mismatch and inadequate information allocation, limiting multi-scale feature representation and weakening the model’s adaptability to defects at different scales, thereby impairing overall detection performance. To address these issues, we propose an edge–discrepancy collaborative defect detection method for casting DR images, namely MTS-YOLOv11: (1) To tackle the problem of “weak edge details of small defects that easily decay with downsampling,” we introduce a Multi-Scale Edge Information Enhancement module (MSEES) into the backbone to amplify high-frequency edge and texture responses and improve the perceptibility of tiny defects; (2) to address “insufficient dependency modeling under complex backgrounds and multi-defect coexistence,” we embed TripletAttention in high-level backbone stages to jointly optimize channel and spatial dependencies, enhancing defect responses while suppressing background interference; (3) to mitigate “scale mismatch and background information leakage during shallow–deep fusion,” we design a Scale-Discrepancy-Aware Gated Fusion module (SDAGFusion) before the detection head to explicitly coordinate complementary multi-scale features, enabling high-accuracy and robust detection of pores, inclusions, and looseness. Importantly, MTS-YOLOv11 is not a simple plug-in combination of generic attention modules or multi-scale fusion blocks. Instead, it is built around the key bottlenecks observed in casting DR defect detection-weak edge cues, pronounced scale discrepancy, and strong interference from complex background textures, thus adopting an edge–discrepancy collaborative design. Specifically, MSEES reinforces faint edge and texture details at the early feature-extraction stage to improve the perceptibility of tiny defects; TripletAttention suppresses non-defect activations induced by complex backgrounds in high-level features, reducing false positives and alleviating localization drift; and SDAGFusion dynamically allocates and fuses cross-scale information through a discrepancy-aware gating mechanism, forming a coherent pipeline (edge enhancement–dependency calibration–discrepancy-aware fusion). This synergistic combination distinguishes our method from prior YOLO variants that merely introduce attention blocks or adopt generic multi-scale fusion strategies. The principal contributions of this work are as follows:

1. We propose MTS-YOLOv11—a dedicated detection architecture for casting DR imagery—designed to jointly address the core challenges of small-scale defect localization, low-contrast discrimination, and background heterogeneity. Structural modifications are systematically integrated across the backbone, neck, and head to enhance feature fidelity, contextual coherence, and detection sensitivity, while keeping the computational overhead within a practical range for industrial deployment.

2. A Multi-Scale Edge Information Enhancement System (MSEES) is embedded within the C3K2 module of the backbone. By amplifying high-frequency edge gradients and textural discontinuities in shallow-layer feature maps, MSEES enhances edge/detail cues for small-scale defects, thereby improving early-stage feature perceptibility and reducing fine-detail loss during subsequent downsampling.

3. A TripletAttention mechanism is deployed in the high-level backbone stages to better model channel–spatial dependencies under complex metallurgical textures. By calibrating feature responses along channel–height, channel–width, and spatial dimensions, TripletAttention mainly helps suppress background-induced activations and reduce false positives, thereby improving detection robustness and alleviating localization drift, particularly for inclusion defects that are easily confused with noise patterns.

4. A Scale-Discrepancy-Aware Gated Fusion (SDAGFusion) module is inserted immediately before the detection head to explicitly address scale mismatch during shallow–deep feature fusion. Through a discrepancy-aware gating strategy, SDAGFusion adaptively allocates complementary multi-scale information while suppressing redundant background features, leading to more coherent cross-scale representations and enhanced sensitivity to small, low-contrast defects.

The remainder of this paper is organized as follows. Section 2 details the MTS-YOLOv11 framework, beginning with a concise overview of the baseline YOLOv11 architecture and followed by principled descriptions of the three novel modules—MSEES, TripletAttention, and SDAGFusion—including their architectural rationale, integration points, and functional objectives. Section 3 presents comprehensive experimental validation: quantitative comparisons against eleven state-of-the-art detectors on a standardized casting DR benchmark, ablation studies verifying module efficacy, and qualitative visualizations demonstrating enhanced localization precision and background rejection capability. Section 4 concludes the work, summarizes key technical insights, and discusses practical implications for industrial inline inspection systems.

## 2. Methodology

### 2.1. MTS-YOLOv11 Model Architecture

**Figure 1:**
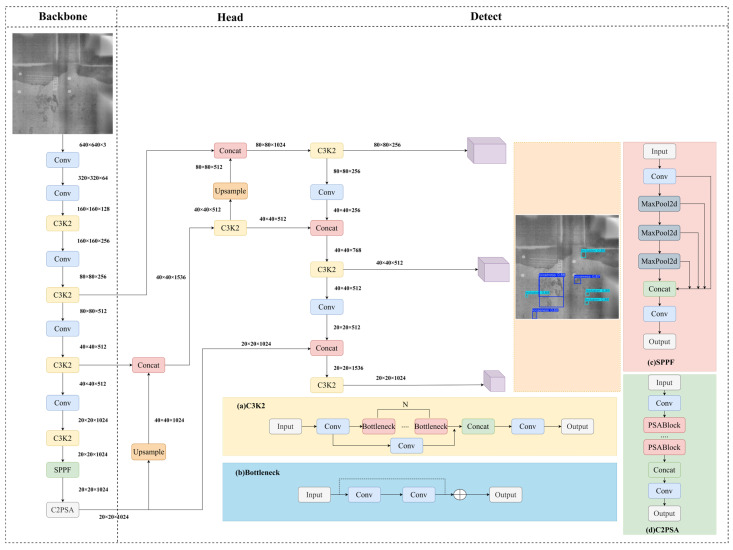
YOLOv11 architecture diagram.

YOLOv11 inherits the core advantages of one-stage detection—namely, end-to-end differentiability, streamlined inference pipelines, and hardware-efficient deployment—while introducing targeted architectural refinements to enhance feature expressiveness and multi-scale reasoning. As illustrated in Figure 1, the architecture comprises three functionally distinct components: Backbone, Neck, and Head. The Backbone extracts hierarchical features from raw input images through a sequence of convolutional layers and C3K2 modules, which jointly perform progressive spatial downsampling and semantic abstraction. Critically, the C3K2 module achieves superior feature representation capacity with minimal parameter overhead—striking an optimal balance between modeling power and computational efficiency. Following the backbone, the SPPF (Spatial Pyramid Pooling Fast) module applies parallel multi-scale max-pooling operations to significantly expand the effective receptive field, thereby enriching feature maps with robust global context and improving sensitivity to spatially dispersed or irregularly shaped defects. To further refine spatial attention, the C2PSA (Cross-Stage Partial Spatial Attention) module is integrated into later backbone stages, dynamically recalibrating feature responses along spatial dimensions while preserving channel-wise discriminability. The Neck implements an enhanced Feature Pyramid Network (FPN) that fuses multi-level features via top-down lateral connections, upsampled feature aggregation, and lightweight C3K2 refinement—enabling precise alignment of semantic richness and spatial fidelity across scales, which is essential for detecting small defects embedded in heterogeneous backgrounds. Finally, the Head adopts a fully decoupled structure: separate convolutional branches for classification and bounding-box regression, each optimized with task-specific normalization and activation schemes. This architectural separation not only accelerates training convergence but also mitigates gradient interference between tasks, leading to demonstrably improved localization precision and class confidence calibration.

**Figure 2:**
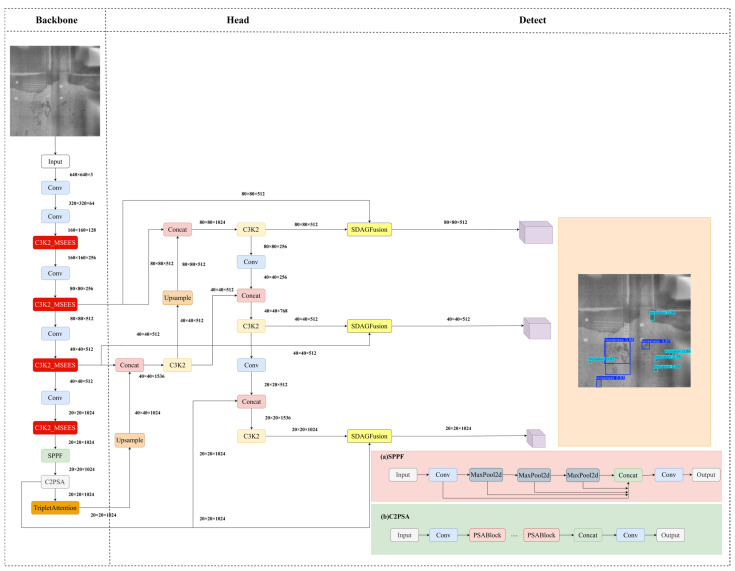
MTS-YOLOv11 architecture diagram.

To address the persistent challenges in detecting casting defects—including pores, inclusions, and looseness—in DR imagery, characterized by sub-pixel spatial extent, high morphological diversity, and low contrast-to-noise ratio (CNR), this study introduces structural refinements to the YOLOv11 architecture, yielding the MTS-YOLOv11 framework. As illustrated in Figure 2, the optimized architecture incorporates three synergistic enhancements: First, the C3K2 module in the backbone is augmented with a Multi-Scale Edge Information Enhancement System (MSEES), which injects multi-scale edge-aware priors into shallow feature maps via parallel convolutional paths of varying kernel sizes and dilation rates. This preserves geometrically salient high-frequency cues—critical for resolving minute defect boundaries—while enabling hierarchical edge-feature fusion without introducing significant computational latency. Second, a TripletAttention mechanism is integrated into the high-level backbone stages to jointly recalibrate feature responses along channel–height, channel–width, and native spatial dimensions. By modeling inter-dimensional dependencies in a unified attention space, it selectively amplifies activations at true defect locations while suppressing spurious responses from metallurgical texture noise and background clutter. Third, a Scale-Discrepancy-Aware Gated Fusion (SDAGFusion) module is deployed immediately before the detection head. SDAGFusion explicitly quantifies scale-level discrepancies—including resolution mismatch, semantic abstraction gap, and feature magnitude variance—through learnable gating functions, then applies pixel-wise adaptive weighting to cross-scale feature maps. This dynamic fusion strategy effectively suppresses background redundancy, enhances spatial coherence of defect representations, and ensures robust responsiveness to both low-CNR anomalies and morphologically ambiguous structures.

### 2.2. MSEES Model

**Figure 3:**
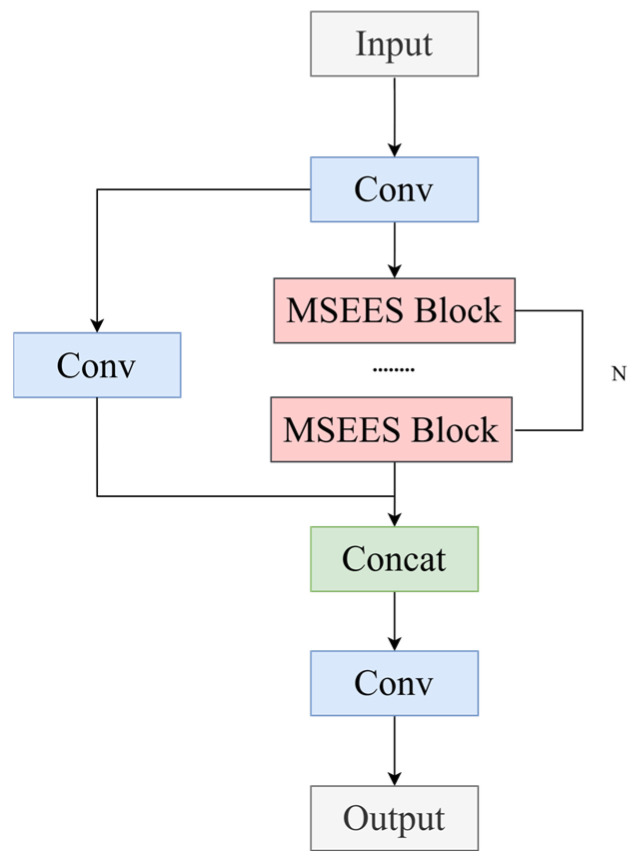
C3K2_MSEES.

**Figure 4:**
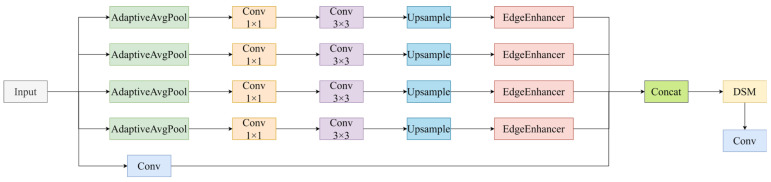
MSEES Module.

Given the inherent scale variability and morphological heterogeneity of casting defects in DR imagery—ranging from sub-pixel pores clusters to irregularly shaped inclusions—the model’s capacity for multi-scale feature perception is a fundamental prerequisite for robust detection. To this end, we redesign the C3K2 module in the backbone by replacing its core Bottleneck block with a Multi-Scale Edge Information Enhancement and Selection (MSEES) module, yielding the enhanced C3K2_MSEES unit (Figure 3). MSEES comprises three tightly coupled components: (1) a parallel multi-scale convolutional pathway for hierarchical edge-aware feature extraction; (2) an edge enhancement branch that applies high-pass filtering and gradient magnitude normalization to amplify geometrically salient boundary cues; (3) a Dual-domain Selection Mechanism (DSM) that jointly performs channel-wise attention and spatial soft-gating to suppress background-irrelevant activations while preserving discriminative edge structures. Critically, MSEES operates exclusively within shallow layers—where high-frequency detail is most abundant—and introduces negligible computational overhead, ensuring compatibility with real-time industrial inspection constraints. By unifying multi-scale contextual modeling, edge-specific enhancement, and domain-adaptive selection, MSEES effectively filters out redundant or noise-corrupted edge responses and delivers compact, high-fidelity feature representations enriched with geometrically grounded defect signatures to downstream network stages. The complete architectural specification of MSEES is provided in Figure 4.

Specifically, in the MSEES module, the input feature is first processed by adaptive average pooling at multiple scales, so that features can be extracted under different receptive fields to obtain multi-scale feature representations. Let the input feature be denoted as $X\in R^{C\times H\times W}$, where H and W are the height and width, respectively. After applying pooling operations with different scales, the output feature at each scale s can be expressed as Equation (1):

(1) $$F_{i}={\mathrm{Pool}}_{i}(X)$$

Then, for each scale, the corresponding feature map is first passed through a 1×1 convolution to perform channel compression, followed by a depthwise convolution to extract local features. After these convolutions, the feature at each scale is upsampled back to the original spatial resolution to maintain a consistent size, which can be expressed as Equation (2):

(2) $$F_{i}^{\prime \prime }=\mathrm{Upsample}\left(\mathrm{Conv}\left({\mathrm{DWConv}}_{3\times 3}\left({\mathrm{Conv}}_{1}\left(F_{i}\right)\right)\right),(H,W)\right)$$

In Equation (2), ${\mathrm{Conv}}_{1}\left(F_{i}\right)$ denotes the 1×1 convolution applied to the i-th feature map, and DWConv(⋅) denotes the depthwise separable convolution applied to each scale-specific feature map.

**Figure 5:**
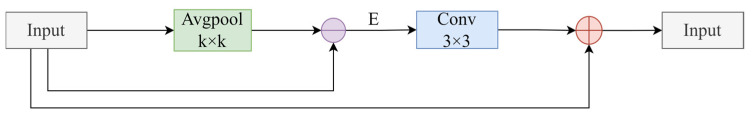
Edge Enhancement Module.

After multi-scale feature extraction, an edge enhancement operation is further applied to strengthen the response to high-frequency components and object boundaries by using low-frequency smoothing and high-frequency differencing to extract edge features. Specifically, an average pooling operation with kernel size k×k is used to obtain the low-frequency component of the input feature map, yielding a low-frequency feature map. The high-frequency information E is then obtained by subtracting this low-frequency feature map from the original feature map X. Subsequently, E is passed through a 3×3 convolution layer followed by a Sigmoid activation function to perform nonlinear enhancement of the high-frequency features, resulting in an enhanced edge feature map. Finally, the enhanced edge feature map is added back to the original feature map X to form the output. The structure of this edge enhancement branch is illustrated in Figure 5.

Subsequently, to further optimize the fusion of features at different scales, a Dual-domain Selection Mechanism (DSM) is introduced, which adaptively weights features in both the channel and spatial domains to reduce redundant information and highlight important regions. Specifically, the multi-scale features are first concatenated as follows:

(3) $$F_{\mathrm{cat}\;}=\mathrm{Concat}\left(F_{1}^{\prime \prime },F_{2}^{\prime \prime },\ldots ,F_{n}^{\prime \prime }\right)$$

Direct concatenation may lead to competition among channels and redundant spatial responses, so an adaptive weighting mechanism is introduced to refine and optimize the fused features. First, in the channel domain selection branch, each channel in the feature map can be regarded as a semantic subspace perceived by the network, representing the response strength to specific categories or textures. To model the correlation among channels, a channel attention map $A_{c}$ is computed. Specifically, global average pooling is applied to compress the spatial dimensions, allowing the network to focus on the global semantic distribution:

(4) $$A_{c}=\sigma \left(\mathrm{FC}\left(\mathrm{GlobalAvgPool}\left(F_{\mathrm{cat}}\right)\right)\right)$$

In Equation (4), FC(⋅) denotes the fully connected layer, which performs a linear transformation, and $\mathrm{GlobalAvgPool}\left(F_{\mathrm{cat}}\right)$ denotes the global average pooling applied to $F_{\mathrm{cat}}$.

In the spatial domain, the module captures local response patterns through a spatial attention map to highlight important spatial regions. The spatial attention map is computed as follows:

(5) $$A_{s}=\sigma \left(\mathrm{Conv}\left(\mathrm{AvgPool}\left(F_{\mathrm{cat}}\right),\mathrm{MaxPool}\left(F_{\mathrm{cat}}\right)\right)\right)$$

In Equation (5), $\mathrm{MaxPool}\left(F_{\mathrm{cat}}\right)$ denotes the max pooling operation, and σ denotes the Sigmoid activation function.

Finally, the features weighted by channel and spatial attention are fused and passed through a convolution layer to obtain the final output:

(6) $$F_{\mathrm{output}\;}=\mathrm{Conv}\left(A_{s}\cdot A_{c}\cdot F_{\mathrm{cat}\;}\right)$$

### 2.3. TripletAttention Mechanism

To address the limitations of the backbone network in detailed feature representation and suppression of irrelevant information, a TripletAttention mechanism is introduced into the high-level stages of the backbone. This module takes the convolutional feature map $X\in R^{C\times H\times W}$ as input and performs feature re-arrangement and weighting along different dimensions to jointly model channel and spatial dependencies. TripletAttention adopts a three-branch parallel structure. In the first branch, the original layout is preserved, and the feature map is fed into the AttentionGate in the form (C,H,W) to capture conventional channel–spatial dependencies. By adaptively adjusting the importance of different regions or channels in the input feature map, this branch enables the network to focus on task-relevant regions while suppressing irrelevant areas. In the second branch, the height dimension H is re-arranged to a channel-like position to form a feature $X_{HCW}$, which emphasizes height–channel relationships; in this case, the AttentionGate treats the height dimension as a channel dimension for weighting. In the third branch, the width dimension W is re-arranged to a channel-like position to form a feature $X_{WHC}$, emphasizing width–channel relationships. The three branches are each passed through an identical AttentionGate sub-module, which adaptively learns important channels and spatial positions from their respective perspectives and produces weighted intermediate representations. After attention weighting, the second and third branches are restored to the unified (C,H,W) layout via inverse dimension re-arrangement, while the first branch remains unchanged; the outputs of the three branches are denoted as $\tilde{X_{1}}$, $\tilde{X_{2}}$, and $\tilde{X_{3}}$, respectively. Finally, these three outputs are fused element-wise to obtain the attention-enhanced feature:

(7) $$Y=\left\{\begin{array}{l} \frac{1}{3}\left(\tilde{X_{1}}+\tilde{X_{2}}+\tilde{X_{3}}\right)\;\\ \frac{1}{2}\left(\tilde{X_{2}}+\tilde{X_{3}}\right)\; \end{array}\right.$$

**Figure 6:**
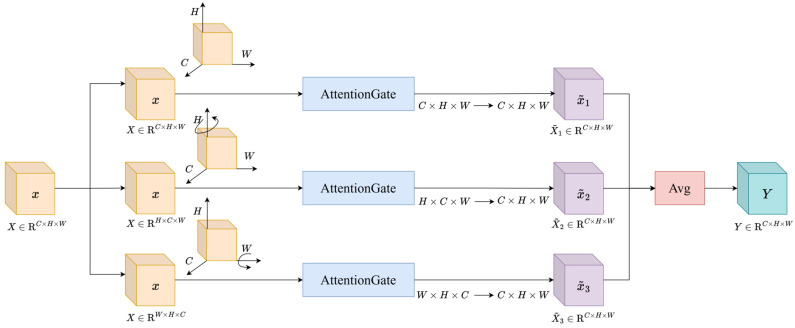
TripletAttention Module.

The structure of the TripletAttention module is shown in Figure 6. By jointly modeling features from three complementary perspectives—height–channel, width–channel, and the original spatial domain—TripletAttention can effectively capture cross-dimensional dependencies and structural relationships, thereby strengthening the response to target regions while suppressing background interference.

### 2.4. SDAGFusion Module

To effectively alleviate the problems of semantic inconsistency and spatial information imbalance that arise during multi-layer feature fusion for defects of different scales in casting DR images, a Scale-Discrepancy-Aware Gated Fusion module (SDAGFusion) is designed at the front of the detection head. This module fuses shallow and deep features while explicitly modeling the scale discrepancy between them. By jointly leveraging channel attention, spatial attention, and pixel-wise gating mechanisms, it constrains the fusion process from three complementary perspectives: semantic selection, spatial localization, and local detail modulation. Benefiting from the combination of scale-discrepancy modeling and gated adaptive fusion, the network can adaptively highlight defect-related key responses and suppress background noise and redundant information when integrating multi-scale features. As a result, global semantic consistency is preserved while the spatial distinguishability and pixel-level representation of defect regions are further enhanced, providing more discriminative feature inputs for the subsequent classification and localization heads.

**Figure 7:**
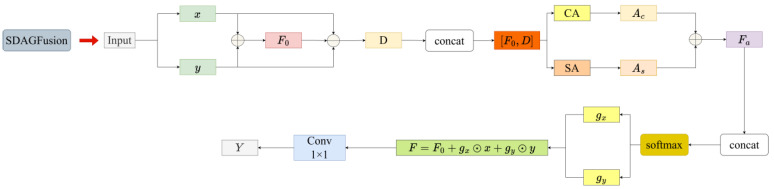
SDAGFusion Processing Procedure.

Specifically, the SDAGFusion module simultaneously receives two input features x and y from different levels, representing shallow and deep features, respectively. The shallow feature has a higher spatial resolution and contains rich edge and texture details, whereas the deep feature carries stronger semantic information but weaker spatial details. The two inputs are first added element-wise to obtain an initial fused feature $F_{0}$, which achieves a preliminary integration of semantic and spatial information. At the same time, a scale-discrepancy feature D=|x-y| is constructed to explicitly characterize the response difference between shallow and deep features in defect and background regions. Next, $\left[F_{0},D\right]$ are concatenated along the channel dimension and fed into the channel attention and spatial attention modules to obtain the channel attention map $A_{c}$ and the spatial attention map $A_{s}$, respectively. These two maps are then added to form a cross-scale relation feature $F_{\alpha }$, which encodes the global and local weighting patterns of the fused features. On this basis, $F_{0}$ and $F_{\alpha }$ are concatenated again and passed through a lightweight convolutional subnetwork to generate a two-channel gating map. After Softmax normalization, pixel-wise weighting coefficients $g_{x}$ and $g_{y}$ corresponding to the shallow and deep branches are obtained. The final fused feature is then computed by combining x and y under these learned gates together with the initial fusion F. Finally, a 1×1 convolution is applied to F to further compress the channel dimension and complete feature integration, yielding the output feature Y. The overall structure of SDAGFusion is illustrated in Figure 7.

## 3. Experiments and Results

### 3.1. Experimental Platform and Parameter Settings

The experiments were conducted on a Windows 10 operating system. The hardware configuration consisted of an Intel (R) Core (TM) i5-11400 @ 2.60 GHz CPU, an NVIDIA GeForce RTX 3060 GPU, and 16 GB of RAM. To ensure the accuracy and stability of the experimental results, the deep learning framework PyTorch 2.2.2 was adopted with Python 3.10.14 as the programming language, and CUDA 12.1 was used to enable GPU-accelerated computation.

To guarantee the effectiveness and fairness of the comparative experiments, all models were trained with identical key hyperparameter settings, thereby avoiding performance discrepancies caused by configuration differences. In particular, the input resolution was fixed to 640 × 640 pixels for all methods; the number of training epochs was set to 300, and the batch size was fixed at 16, ensuring that each model was evaluated under the same data iteration conditions. In addition, the initial learning rate was set to 0.01, the momentum factor to 0.937, and the optimizer was consistently chosen as SGD with a weight decay of 0.0005 and an early-stopping patience of 100 epochs. These unified configurations ensure that comparisons among different models are made on a common basis, enhancing the credibility and reproducibility of the experimental results..

### 3.2. Casting DR Image Defect Dataset

**Figure 8:**
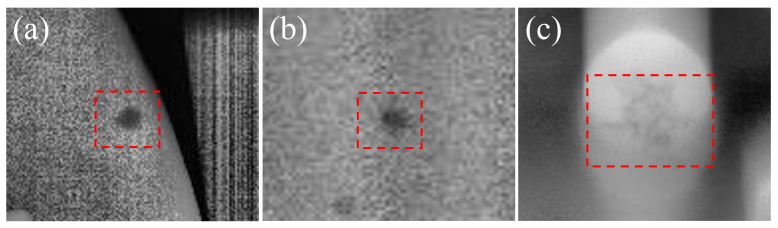
Casting Defects: (**a**) pore; (**b**) inclusion; (**c**) looseness.

The dataset used in this study is derived from X-ray DR images of cast components provided by Qingdao Sanheshan Precision Casting Co., Ltd. (Qingdao, China). All images were acquired on a fixed industrial DR inspection line operated under the company’s standard non-destructive testing (NDT) procedure. All inspections were carried out in a controlled workshop environment under normal ambient temperature and humidity, and no manual post-processing beyond the DR system’s built-in correction pipeline was applied. For confidentiality reasons, the manufacturer does not disclose detailed hardware specifications; however, the same system and acquisition settings were kept constant throughout data collection, ensuring consistent imaging conditions across all samples. It contains three typical types of defects: pore, inclusion, and looseness, as illustrated in Figure 8. Pore defects are caused by gas that fails to escape in time during the casting process and becomes trapped as the metal solidifies, forming closed cavities. Their sizes and spatial distributions are diverse, and they can appear in any region of the casting; in DR images, they usually exhibit relatively regular shapes and appear as dark circular or near-circular regions with approximately uniform distribution. Inclusion defects originate from slag or metal oxides that are not completely removed during pouring and remain inside the casting, forming heterogeneous phases. In DR images, inclusions typically appear as dark irregular polygons or cluttered shapes with relatively low boundary sharpness. Looseness defects result from locally non-uniform cooling of the molten metal, where looseness or gas evolution leads to clusters of small voids. In DR images, looseness generally manifests as dendritic, honeycomb-like, or sponge-like textures with pronounced spatial connectivity and structural characteristics.

**Figure 9:**
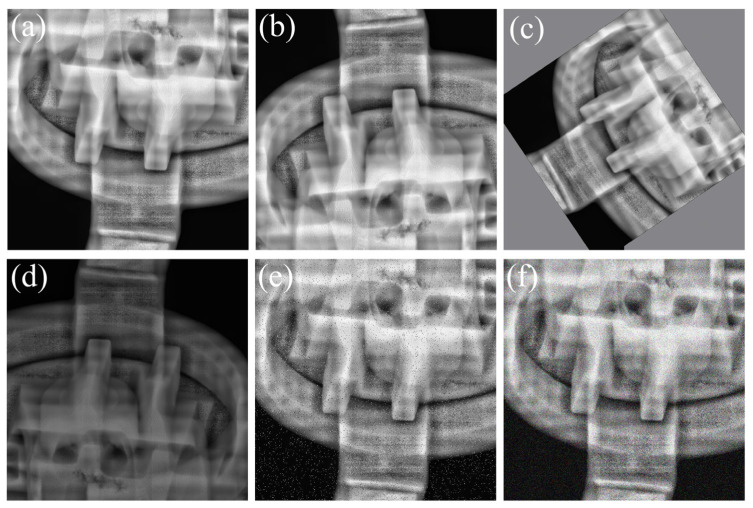
Data augmentation schematic diagram. (**a**) Original image; (**b**) Flip; (**c**) Selection; (**d**) Brightness and darkness variation; (**e**) Salt and pepper noise; (**f**) Gaussian noise.

**Figure 10:**
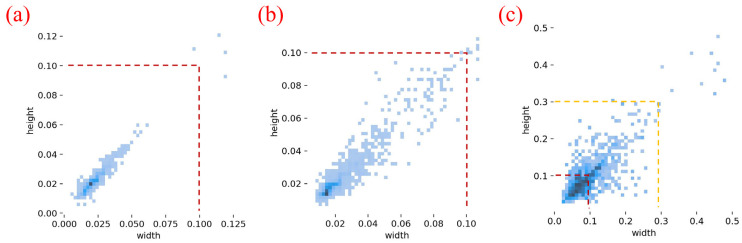
Proportion of defect sizes. (**a**) Pore; (**b**) Inclusion; (**c**) Looseness.

Since the original casting DR images have varying resolutions, all images were first cropped to 640×640 pixels to avoid interference with detection performance after being fed into the model. In addition, this cropping helps preserve sufficient spatial resolution for tiny, densely distributed defects: if full-size DR images were directly downsampled by the backbone, many small defect regions would shrink to only a few pixels or even vanish in deeper feature maps, making them difficult to detect reliably. The images were then screened, and only sub-images containing defects were retained to construct the dataset. The resulting raw dataset consists of 872 casting DR images, which were split into training, validation, and test sets at a ratio of 6:2:2. Because DR image acquisition is costly and defect samples are difficult to obtain, the collected raw data are usually limited in scale, which may adversely affect the robustness and generalization ability of the model. Therefore, without altering the true spatial distribution characteristics of defect targets, multiple data augmentation strategies—including rotation, horizontal flipping, brightness variation, and noise injection—were applied to the partitioned raw dataset to expand the sample size. Representative augmentation results are shown in Figure 9, and a total of 6159 image samples were ultimately generated. Defect annotation was subsequently performed using the LabelImg tool (Version 1.8.6), resulting in the final casting DR image defect dataset. The dataset contains 5562 labeled pore instances, 9748 labeled inclusion instances, and 5430 labeled looseness instances, which were used for model training and final performance evaluation. As shown in Figure 10 (bounding-box width–height distribution), pore and inclusion instances are dominated by small-scale boxes (normalized width/height largely below 0.1), whereas looseness spans a broader range of target sizes with a heavier tail toward larger extents. This small-target dominance and scale variation increase detection difficulty by amplifying fine-detail loss during downsampling and making multi-scale feature fusion more demanding, thereby requiring stronger edge/detail preservation and more reliable cross-scale representations. The adopted dataset partitioning and augmentation strategies further enrich the diversity of pore, inclusion, and looseness defects under different poses, locations, and imaging conditions, which helps improve sample coverage and reduce the risk of overfitting under limited raw data.

### 3.3. Comparative Experiments

In the casting DR image defect detection task, the proposed MTS-YOLOv11 model was comparatively evaluated against several mainstream object detection algorithms. A comprehensive comparison was conducted using multiple metrics, including Precision, Recall, F1-score, mean Average Precision (mAP@0.5 and mAP@0.5:0.95), the number of parameters (Params), and computational complexity (FLOPs). In object detection, a prediction is regarded as a true positive (*TP*) when the Intersection over Union (IoU) between the predicted bounding box and the ground-truth box exceeds a predefined threshold and the predicted category is correct. A false positive (*FP*) occurs when a defect is predicted but no corresponding defect region exists in the ground truth, whereas a false negative (*FN*) refers to a ground-truth defect that is not successfully detected. Based on these definitions, Precision, Recall, and F1-score are calculated as follows:

(8) $$\;\mathrm{Precision}\;=\frac{TP}{TP+FP}$$

(9) $$\mathrm{Recall}\;=\frac{TP}{TP+FN}$$

(10) $$F_{1}=\frac{2PR}{P+R}$$

Here, Precision indicates the proportion of predicted defect samples that are truly defects, Recall measures the proportion of all ground-truth defect samples that are successfully detected, and the F1-score provides a comprehensive assessment of the trade-off between Precision and Recall. A higher value of these metrics generally implies better detection performance.

The improved MTS-YOLOv11 model was compared with several mainstream object detection algorithms, and the precision and recall results for the three defect categories are reported in Table 1. The results indicate that YOLOv11 achieves better overall performance than YOLOv7, YOLOv8, and YOLOv12 on pore, inclusion, and looseness defects, with higher precision and recall, suggesting stronger feature representation capability on casting DR images. Building upon this baseline, the proposed MTS-YOLOv11 further improves detection performance across all three defect types. For pore defects, the precision and recall reach 91.9% and 90.2%, respectively, representing improvements of 2.6 and 4.6 percentage points over the original YOLOv11. For inclusion defects, the precision and recall are 86.7% and 88.9%, respectively, exceeding the baseline by 2.8 and 2.5 percentage points. For looseness defects, the proposed model maintains an almost 100% recall while also increasing precision by 0.6 percentage points.

To further evaluate the overall performance, an analysis based on the F1-score was conducted, and the results are presented in Table 2. As shown in Table 2, the improved MTS-YOLOv11 achieves the highest F1-scores among all compared models for all three defect categories, indicating the best overall performance. Specifically, the F1-scores for pore and inclusion defects increase by 3.6% and 2.7% over the original YOLOv11, respectively, demonstrating that the proposed improvements enhance precision while effectively maintaining recall, thereby achieving a better balance between the two. The F1-score for looseness defects also improves by 0.4%, which further reduces the risks of false detections and missed detections while preserving both high precision and high recall. Taken together with the comparisons of precision and recall, these results confirm that the proposed MTS-YOLOv11 model provides more stable and well-balanced detection capability.

To more comprehensively evaluate the detection performance of each model across different defect categories, the average precision (AP) for the three defect types as well as the overall mAP were further computed and compared. AP provides an integrated measure of the overall performance of a specific defect category by considering the precision–recall relationship under a given IoU criterion. The mean average precision (mAP) is obtained by taking the arithmetic mean of AP over all defect categories, and it is widely used to assess the overall detection capability of a model. When AP is calculated for each category at a fixed IoU threshold of 0.5 and then averaged, the resulting metric is denoted as mAP@0.5. When mAP is computed at multiple IoU thresholds ranging from 0.5 to 0.95 with a step size of 0.05 and then averaged, the metric is referred to as mAP@0.5:0.95, which more comprehensively reflects the stability of the model under different localization accuracy requirements. In addition, the number of parameters (Params) and the computational complexity (FLOPs) are reported to evaluate the resource consumption and computational efficiency of each model. Whereas the inference speed, measured in frames per second (FPS), is used to quantify real-time performance, with higher FPS indicating faster inference. The experimental results are summarized in Table 3.

Table 3 shows that the improved MTS-YOLOv11 model achieves the best performance across all evaluation metrics. Compared with the original YOLOv11 model, the average precision (AP) for pores and inclusions increases to 95.8% and 94.1%, respectively, while the AP for looseness remains at 99.5%. The overall mean average precision mAP@0.5 is improved to 96.5%, and mAP@0.5:0.95 increases from 67.3% to 68.5%. This fully demonstrates that the improved MTS-YOLOv11 model possesses higher detection accuracy and stronger robustness. From the perspective of model complexity, the number of parameters and the computational cost of MTS-YOLOv11 are slightly increased compared with the original YOLOv11. This increase is mainly due to embedding the MSEES module into the improved C3K2 block in the backbone, introducing the TripletAttention mechanism into the higher backbone stages and adding the SDAGFusion module at the front of the detection head. These three modules play roles in local edge modeling, channel–spatial correlation modeling, and multi-scale feature adaptive fusion, respectively. Moreover, all of the above modules adopt designs such as channel splitting and depthwise separable convolutions and are only activated at a few critical stages. As a result, the total number of parameters remains below 3 M and the computational cost stays below 8 G. Compared with the gains in mAP@0.5 and mAP@0.5:0.95, this slight increase in computation is acceptable and necessary. On the same hardware platform, the end-to-end inference speed of MTS-YOLOv11 reaches 359.07 FPS, which is comparable to and slightly higher than the 346.86 FPS of the baseline YOLOv11, indicating that real-time performance is preserved. Overall, MTS-YOLOv11 provides a stable improvement in detection accuracy and robustness for complex casting defects while maintaining a favorable accuracy–efficiency trade-off suitable for industrial deployment.

### 3.4. Ablation Experiments

To further analyze the contributions of each improved module to the overall detection performance, a series of combinational ablation experiments were conducted. Taking the original YOLOv11 model as the baseline, the proposed modules were gradually introduced to systematically evaluate their impact on detection performance. Table 4 reports the per-class AP results (Pore, Inclusion, and Looseness) for each experimental group, enabling a fine-grained comparison of how each module influences category-specific detection performance. Table 5 summarizes the statistical results of each experimental group in terms of mAP@0.5 and mAP@0.5:0.95. Among them, Group 0 corresponds to the baseline YOLOv11 model without any integrated improvement modules and is used as a reference for comparison with the other experimental groups.

As shown in Table 4, the different configurations exhibit noticeable differences in detection performance across the three defect types: pore, inclusion, and looseness. For the baseline model (Group 0), the AP values for these three categories are 94.3%, 91.8%, and 99.5%, respectively, where looseness is already close to saturation and thus less sensitive to subsequent improvements. When only MSEES is introduced (Group 1), the AP for pore and inclusion increases to 95.6% and 93.6%, respectively, while looseness remains at 99.5%. This indicates that pore and inclusion—both highly sensitive to edge and high-frequency detail—are the two defect types that benefit most directly from MSEES, with inclusion showing the largest relative improvement. When only SDAGFusion is added (Group 2), the AP values for pore and inclusion reach 95.2% and 92.7%, again with a more pronounced gain for inclusion. This suggests that scale-discrepancy-aware fusion is particularly effective for inclusion, whose morphology is more variable and whose scale spans a wider range. With only TripletAttention added (Group 3), the AP for pore is 95% and that for inclusion increases to 92.1%; although the overall improvement is modest, it is mainly reflected in the inclusion category, which is more prone to confusion with background noise under complex texture conditions. For pairwise combinations, Group 4 (MSEES + SDAGFusion) further increases the AP for pore and inclusion to 95.5% and 94%, while Group 5 (MSEES + TripletAttention) raises inclusion AP to 93.2%. Both cases again confirm that inclusion is the most sensitive and most benefited category under structural improvements. When all three modules are enabled (Group 7), the AP values for pore, inclusion, and looseness reach 95.8%, 94.1%, and 99.5%, respectively, with inclusion showing the largest improvement over the baseline, followed by pore, and looseness remaining almost unchanged. Overall, the proposed module combinations yield the most notable gains for pore and inclusion, which are small-scale and low-contrast defects, with inclusion benefiting the most, whereas the impact on the already saturated looseness category is relatively limited.

Table 5 reports the overall performance of each configuration. For the baseline YOLOv11 model (Group 0), mAP@0.5 and mAP@0.5:0.95 are 95.2% and 67.3%, respectively. Among the single-module configurations, MSEES (Group 1) yields the most pronounced improvement, raising these metrics to 96.2% and 67.6%. Combined with the observations from Table 4, this overall gain can be attributed primarily to the simultaneous improvement in AP for pore and inclusion. When only SDAGFusion is added (Group 2), mAP@0.5/mAP@0.5:0.95 increases to 95.8%/67.7%, which is consistent with the finding in Table 4 that inclusion benefits more notably; this indicates that scale-discrepancy-aware fusion is particularly effective in improving the detection of inclusion defects with large scale variation. When only TripletAttention is introduced (Group 3), the changes in overall mAP are relatively small (95.5%/67.3%). Its main contribution lies in suppressing false positives for inclusion under complex background textures, so its effect on global metrics is less prominent than that of MSEES and SDAGFusion. Nevertheless, TripletAttention is retained for its complementary role in improving robustness rather than delivering the largest mAP gain: by modeling channel–height, channel–width, and spatial interactions, it helps disentangle channel–spatial dependencies under metallurgical texture clutter, thereby stabilizing localization and reducing texture-induced spurious responses, particularly for morphologically irregular inclusion defects. For the pairwise configurations, Group 4 (MSEES + SDAGFusion) further boosts the metrics to 96.4%/68.3%, and both Groups 5 and 6 also outperform the baseline, indicating a degree of synergy between the modules: on the one hand, MSEES enhances the perceptibility of small targets; on the other hand, SDAGFusion and TripletAttention provide complementary improvements in scale-aware fusion and background suppression, respectively. Finally, the full model (Group 7) achieves the best overall performance, with mAP@0.5/mAP@0.5:0.95 reaching 96.5%/68.5%. Combined with the category-wise results in Table 4, this overall improvement can be seen to stem mainly from enhanced detection of inclusion and pore, with inclusion being the most sensitive to the structural modifications. This confirms that the proposed edge–discrepancy collaborative design offers a more substantial advantage for hard-to-detect, small-scale, low-contrast defects in casting DR images.

To verify the advantage of the proposed MSEES module over simple edge-aware baselines, two comparison methods with fixed edge priors are further constructed by integrating Sobel-edge and Laplacian-edge into the original YOLOv11, corresponding to Groups 2 and 3, respectively, while Group 0 denotes the original YOLOv11 and Group 1 denotes the configuration with only MSEES inserted. As shown in Table 6, compared with the baseline model (Group 0, mAP@0.5 = 95.2%, with pore/inclusion AP of 94.3%/91.8%), introducing only MSEES (Group 1) increases mAP@0.5 to 96.2% and raises pore and inclusion AP to 95.6% and 93.6%, respectively, while keeping the parameter count at 2.57M and the computational cost at 6.6G FLOPs, with only a moderate reduction in FPS (243.69). In contrast, the Sobel-edge [31] baseline (Group 2) performs slightly worse than the baseline on pore and looseness, and its overall mAP@0.5 is only 95.0%, while the number of parameters and FLOPs increase noticeably to 3.74M and 7.9G, and the FPS drops to 201.19. The Laplacian-edge [32] baseline (Group 3) raises mAP@0.5 to 95.7%, but the gain is still smaller than that of MSEES; moreover, with 2.43M parameters and 6.9G FLOPs, its FPS further decreases to 159.23. Overall, the simple Sobel/Laplacian edge-aware baselines provide only limited and sometimes unstable accuracy improvements while incurring higher computational overhead, whereas MSEES yields more substantial gains for small, low-contrast defects such as pores and inclusions under relatively lightweight overhead, indicating that end-to-end learnable multi-scale edge modeling is superior to fixed edge-based baselines.

### 3.5. Repeatability Verification

To further verify the repeatability and statistical stability of the performance improvements brought by the improved model, additional experiments were conducted under multiple random seed settings. Specifically, under exactly the same experimental platform and training configuration, both the baseline YOLOv11 and the proposed MTS-YOLOv11 were independently trained three times with seeds set to 0, 1, and 2. All experiments were carried out on the same hardware environment and adopted the unified hyperparameter configuration described in Section 3.1, while the data augmentation strategy, dataset partition, and evaluation protocol were kept strictly identical across all runs..

As shown in Table 7, the values in parentheses following each defect category AP and mAP@0.5 denote the ranges obtained from three independent trainings under different random seeds (seed = 0, 1, 2), i.e., (min–max). For the baseline YOLOv11, the APs for pore, inclusion, and looseness lie in the ranges (93.81–94.27), (91.72–92.99), and (99.40–99.50), respectively, while the overall mAP@0.5 falls within (95.06–95.40). The corresponding mean and standard deviation of mAP@0.5 over the three runs are approximately 95.22 ± 0.17 (reported as 95.2 ± 0.2), indicating a certain degree of variation under different random initializations. In contrast, for MTS-YOLOv11, the APs for pore, inclusion, and looseness consistently fall within (95.83–96.27), (93.55–95.62), and (98.38–99.50), respectively, and mAP@0.5 lies in the range (96.30–96.76), with a mean and standard deviation of about 96.51 ± 0.23 (reported as 96.5 ± 0.2). It can be observed that, both in terms of ranges and mean values, MTS-YOLOv11 achieves overall performance clearly superior to YOLOv11 across the three runs, and even its worst result (96.30) still exceeds the best result of YOLOv11 (95.40). At the same time, the standard deviations of the two models are of the same order of magnitude, indicating that the introduction of the improved modules does not lead to increased instability due to the slightly larger model capacity. Therefore, under identical training configurations and data conditions, the proposed method can stably obtain an mAP improvement of about 1–1.3 percentage points across multiple random seeds, thereby confirming the repeatability and statistical stability of the performance gains achieved by MTS-YOLOv11.

### 3.6. Comparison of Visualization Results

**Figure 11:**
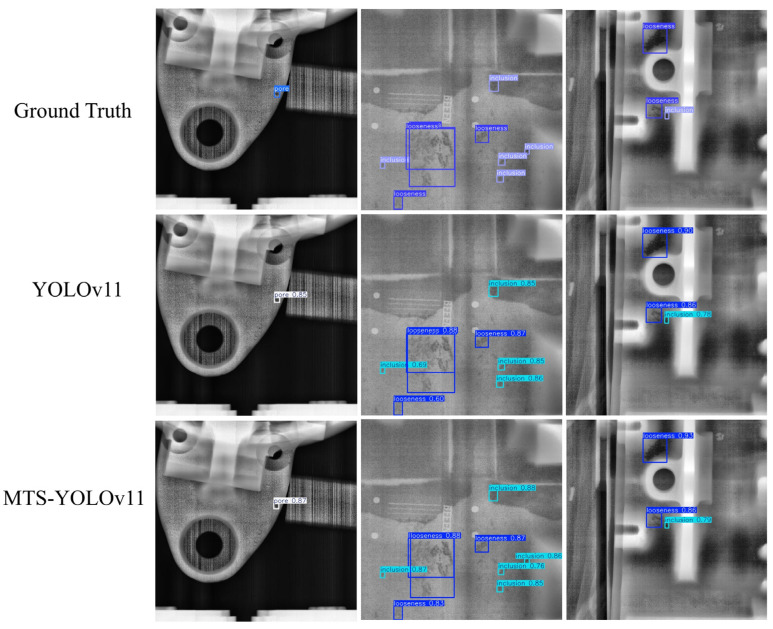
Comparison of model detection effects.

**Figure 12:**
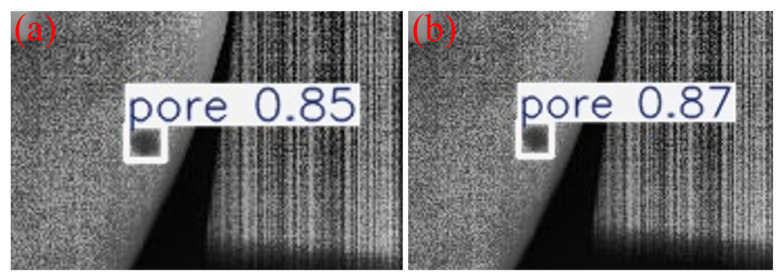
Comparison of detection results for pore defects. (**a**) The detection effect of the original model; (**b**) improved model detection performance.

**Figure 13:**
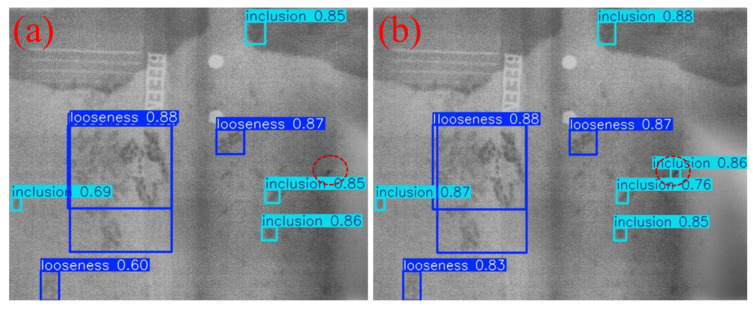
Comparison of detection results for inclusion defects. (**a**) The detection effect of the original model; (**b**) improved model detection performance.

**Figure 14:**
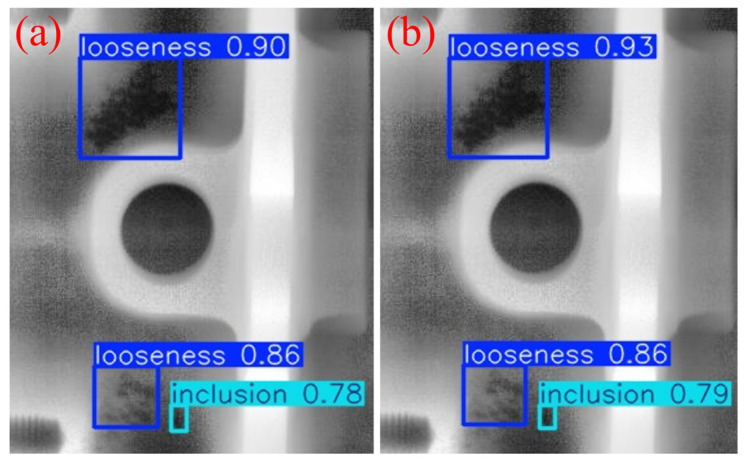
Comparison of detection results for looseness defects. (**a**) The detection effect of the original model; (**b**) improved model detection performance.

To intuitively demonstrate the performance improvement of the proposed model in casting DR image defect detection, Figure 11 presents a visual comparison of the detection results. All images shown in this subsection are sampled from the independent test set under the same evaluation protocol, without any manual cherry-picking. From top to bottom, the rows correspond to the ground-truth annotations (Ground Truth), the detection results of the original YOLOv11 model, and those of the improved MTS-YOLOv11 model, respectively; from left to right, the columns show the detection outputs for pore, inclusion, and looseness defects. Figure 12, Figure 13 and Figure 14 are enlarged local comparisons of the detection effects for pore, inclusion, and looseness defects, respectively, which further highlight the advantages of MTS-YOLOv11 in terms of small-target localization accuracy and boundary recognition clarity.

Based on the visualization results in Figure 11, Figure 12, Figure 13 and Figure 14, both the original YOLOv11 model and the improved MTS-YOLOv11 model are able to identify the three main defect types; however, there are obvious differences in detection quality. The MTS-YOLOv11 model produces higher confidence scores, and its predicted bounding boxes exhibit better overlap with the ground-truth boxes, indicating more stable responses to small-scale defects and stronger localization capability. In particular, in Figure 13, the original YOLOv11 model shows missed detections for inclusion-type defects, where the regions marked by red dashed circles correspond to defect instances that are not detected, whereas the improved MTS-YOLOv11 model can completely and accurately identify all defect targets. In summary, MTS-YOLOv11 outperforms the original model in terms of localization accuracy, confidence outputs, and detection completeness in multi-defect coexistence scenarios.

**Figure 15:**
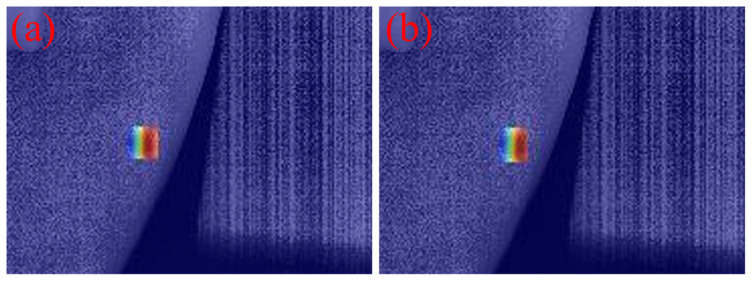
Comparison of heat maps for pore defects. (**a**) Heat map of the original model; (**b**) heat map of the improved model.

**Figure 16:**
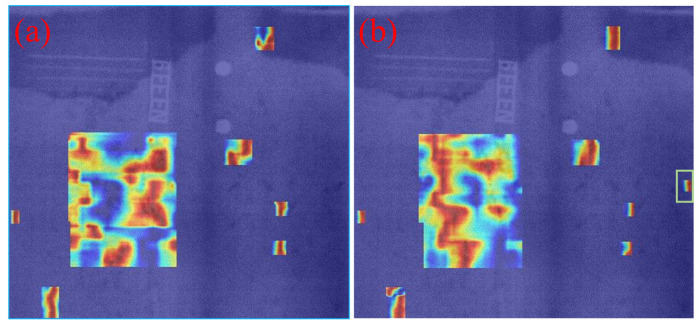
Comparison of heat maps for inclusion defects. (**a**) Heat map of the original model; (**b**) heat map of the improved model.

**Figure 17:**
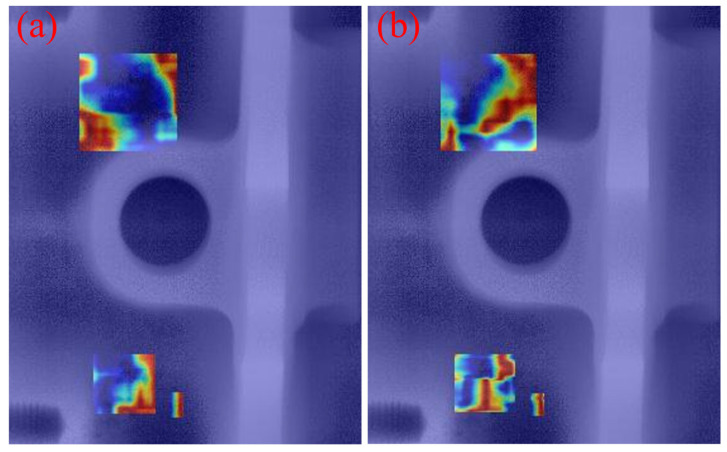
Comparison of heat maps for looseness defects. (**a**) Heat map of the original model; (**b**) heat map of the improved model.

Furthermore, Figure 15, Figure 16 and Figure 17 present comparative attention heatmaps for the three defect types, which further highlight the advantages of the improved model in terms of its ability to focus on defect regions. These heatmaps are obtained using a Grad-CAM-based visualization applied to several high-level neck/head layers of the detector. Concretely, each DR image is first resized to 640 × 640 using the same letterbox pre-processing as in detection and normalized to 0.1; the summed scores of the top 2% highest-confidence detections (confidence > 0.2) are then used as the backpropagation target, and the resulting Grad-CAM response maps are aggregated across channels, with min–max normalized to 0.1, upsampled to the input resolution, and overlaid on the original DR images as pseudo-color heatmaps. In these heatmaps, the color intensity reflects the degree of attention the network pays to different regions of the input image, where brighter colors (such as red or yellow) indicate stronger activation responses and, ideally, should be highly consistent with the actual defect locations. As can be seen, the responses of the improved MTS-YOLOv11 are more concentrated in the pore, inclusion, and looseness defect areas, and the highlighted regions align more tightly with the defect boundaries. This effect is particularly evident for challenging samples with low contrast or very small sizes, where MTS-YOLOv11 is still able to form high-response regions around defect edges and thus significantly reduces the risk of missed detections. By contrast, the original model exhibits weakened or even completely missing attention on some small targets; for example, in Figure 16a, there is a clearly visible region with missed detections. These observations indicate that the MTS-YOLOv11 model has a stronger background suppression capability and can effectively filter out irrelevant noise under complex imaging conditions, thereby achieving more accurate and more complete localization of defect regions.

**Figure 18:**
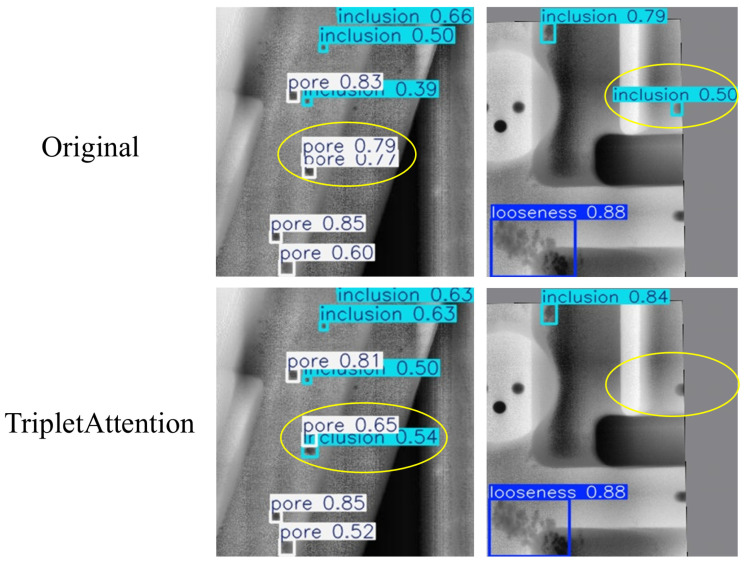
Comparison of the verification results of TripletAttention.

In addition, to qualitatively validate the effect of TripletAttention, Figure 17 presents a comparison of detection results before and after introducing TripletAttention. In complex background regions, the baseline YOLOv11 produces obvious false positives and slightly misaligned bounding boxes for inclusion defects. After TripletAttention is applied, these spurious responses are effectively suppressed and the predicted boxes align more closely with the true defect regions. In Figure 18, false detections are highlighted with yellow circles: in the original YOLOv11 results, some inclusion defects are incorrectly classified as pores, and certain background regions are mistakenly detected as inclusions. With TripletAttention enabled, these errors are successfully corrected and the true defects are accurately detected, which indirectly reflects a substantial improvement in detection accuracy for inclusion defects. This qualitative phenomenon is consistent with the ablation results in Table 4 and Table 5, and it further confirms that TripletAttention mainly enhances overall detection robustness by reducing texture-induced false positives and stabilizing the localization of morphologically irregular, small-scale defects.

**Figure 19:**
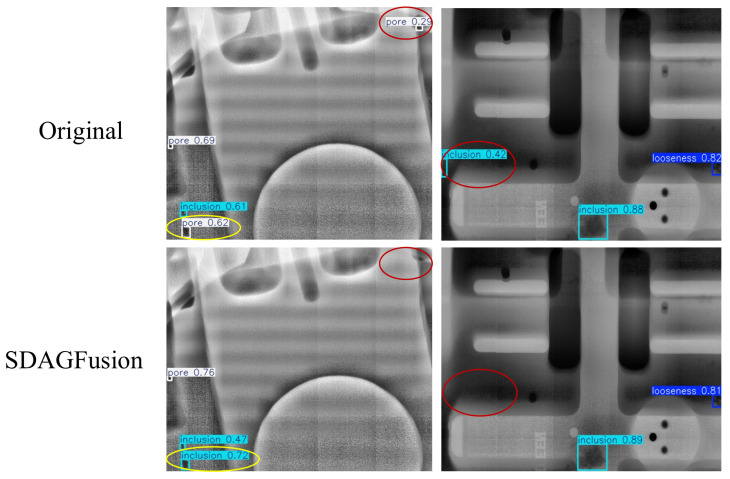
Comparison of the verification results of SDAGFusion.

Furthermore, to qualitatively analyze the behavior of SDAGFusion in defect and background regions, Figure 19 presents a comparison between the detection results of the baseline YOLOv11 (Original) and those of the model with SDAGFusion. In the Original results, the regions highlighted by red circles correspond to background structures that are incorrectly detected as defects, while the yellow circles indicate misclassified targets (e.g., inclusion regions mistakenly predicted as pores). After SDAGFusion is introduced, these spurious responses in background areas are effectively suppressed and are no longer detected as defects, whereas the true defect regions are correctly identified with more reasonable confidence scores. This qualitative evidence shows that the proposed scale-discrepancy-aware gating not only reduces background-induced false positives, but also encourages the network to assign stronger responses to real defect regions. These observations are consistent with the quantitative improvements reported in Table 4 and Table 5, particularly in the enhanced detection performance for inclusion defects under complex background textures.

### 3.7. Analysis of Failure Cases

**Figure 20:**
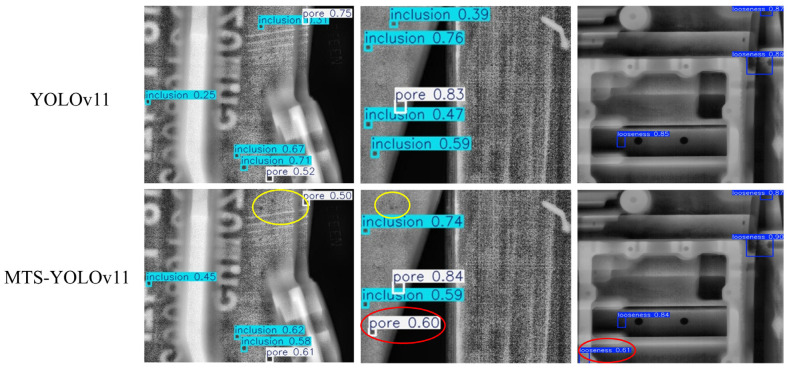
Representative failure cases of YOLOv11 and MTS-YOLOv11 on casting DR images.

To further illustrate that the improved model still has limitations, several representative failure cases from the test set are shown in Figure 20. The top row presents the detection results of YOLOv11, and the bottom row shows those of MTS-YOLOv11. In these examples, yellow circles indicate missed targets (false negatives), while red circles mark false detections or misclassified targets (false positives). These samples generally exhibit strong background textures, extremely small and densely distributed defects, and thus can be regarded as representative hard cases.

As shown in the left and middle columns of Figure 20, even after introducing MTS-YOLOv11, some tiny pores/inclusions adjacent to high-contrast structural edges are still partially missed; for some very low-contrast defects, the model can detect them but with relatively low confidence. In the right-hand example, MTS-YOLOv11 suppresses many spurious detections compared with YOLOv11 and localizes the true looseness regions more accurately, but one looseness region at the bottom (red circle) is still incorrectly detected. These phenomena indicate that, although MTS-YOLOv11 has clearly reduced false positives and false negatives relative to YOLOv11, its detection performance still needs improvement in scenarios involving defects overlapping with complex structural edges and extremely dense clusters of small targets.

Overall, these failure cases show that the proposed edge–discrepancy collaborative design substantially enhances robustness for typical casting DR scenarios, but certain limitations remain under extreme conditions. In future work, instance-level segmentation priors, stronger global context modeling, or uncertainty-aware post-processing could be explored to further improve reliability in real-world foundry inspection environments.

### 3.8. Generalization Experiments

**Figure 21:**
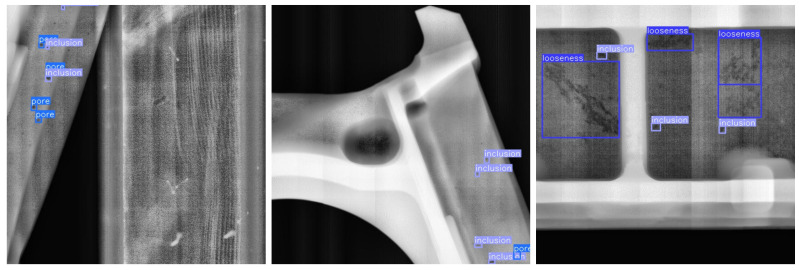
Examples of defects in the new casting DR Image defect dataset.

To further verify the generalization ability of MTS-YOLOv11 in real industrial scenarios, additional DR images were collected on the basis of the original dataset from different production batches and different casting types. The same preprocessing procedure as in Section 3.1 was applied, where all new DR images were uniformly cropped to a size of 640×640 pixels. On this basis, sub-images containing defect regions were extracted according to the defect annotations, resulting in 578 new casting DR images that were used as an independent test set. This dataset differs from the training set in terms of workpiece structure, imaging pose, and background clutter, while still containing the three defect types of pores, inclusions, and looseness, thereby simulating the impact of working-condition variations in actual production on the detection model. Figure 21 illustrates representative defect examples from the new external casting defect dataset.

In the generalization experiments, the same experimental platform and parameter settings as those in Section 3.1 were adopted. The 578 new DR images did not participate in training or validation and were used solely as an external test set. As shown in Table 8, on this new dataset the improved MTS-YOLOv11 model still outperforms the other models overall across all evaluation metrics. In particular, the AP values for pore and inclusion defects reach 92.3% and 84.1%, respectively, both of which are the highest among all methods and correspond to improvements of 3.2 and 0.9 percentage points over the original YOLOv11. The looseness defects also maintain a relatively high AP value. From a category-wise perspective, the performance drop on the new dataset is more pronounced for pores and inclusions than for looseness, which is consistent with their data characteristics: pores and inclusions are typically small-scale and low-contrast, making them more sensitive to changes in alloy composition, part geometry, and imaging conditions, whereas looseness tends to form larger, more contiguous structures whose texture patterns are relatively more stable across domains. Nevertheless, MTS-YOLOv11 consistently yields higher AP for pores and inclusions than YOLOv11 on the new dataset, indicating that the proposed edge–discrepancy collaborative design effectively enhances robustness for these more challenging, domain-sensitive defect types. In terms of aggregated indicators, the overall mean average precision values mAP@0.5 and mAP@0.5:0.95 of MTS-YOLOv11 are 91.3% and 65.4%, respectively, which correspond to improvements of 1.4 and 5.5 percentage points compared with the best baseline model YOLOv11. These results indicate that the proposed MTS-YOLOv11 model can still maintain strong detection accuracy and generalization capability on new casting data.

It should be noted that both datasets were acquired from the same manufacturer and DR imaging system but correspond to different casting batches and different casting types. Therefore, the current generalization study mainly reflects intra-manufacturer domain shifts induced by changes in production batches and part types, rather than cross-vendor or cross-system variations. Due to practical constraints in accessing and sharing industrial DR data, it is currently not feasible to systematically cover samples from multiple manufacturers, heterogeneous DR devices, and broader process conditions. A more systematic evaluation across different manufacturers and imaging systems will be pursued in future works.

## 4. Conclusions

To enhance the detection performance of small-scale, low-contrast defects such as pores, inclusions, and looseness in casting DR images under complex metallurgical backgrounds, this paper proposes MTS-YOLOv11, an edge–discrepancy collaborative detection framework built upon YOLOv11. The method introduces a Multi-Scale Edge Information Enhancement System (MSEES) in shallow backbone stages, a TripletAttention mechanism in high-level features, and a Scale-Discrepancy-Aware Gated Fusion (SDAGFusion) module before the detection head. These three components jointly optimize feature representation from the perspectives of “edge detail enhancement,” “channel–spatial dependency calibration,” and “scale-discrepancy-aware adaptive fusion.” The effectiveness and engineering feasibility of the proposed approach are systematically validated through ablation studies, cross-dataset generalization experiments, inference efficiency analysis, and multi-seed repeatability tests. The main conclusions are as follows:

1. The proposed MSEES module operates in the shallow backbone to amplify geometrically salient edge gradients and textural discontinuities, thereby improving sensitivity to sub-pixel defect boundaries and particularly benefiting the detection of dense pores and fine inclusions. The TripletAttention mechanism, embedded in high-level backbone stages, jointly recalibrates feature responses along three dimensions—channel–height, channel–width, and native spatial space—selectively enhancing activations at true defect locations while suppressing spurious responses induced by grain-boundary noise and complex background textures. This effect is especially pronounced for inclusions, which are morphologically irregular and prone to confusion with the background. The SDAGFusion module is placed immediately before the detection head and explicitly models the differences in spatial resolution and semantic abstraction between shallow and deep features. By performing pixel-wise adaptive reweighting between “detail features” and “semantic features,” SDAGFusion mitigates conflicts across hierarchical representations, suppresses background redundancy, and produces more spatially coherent and discriminative fused features, providing more reliable inputs for subsequent classification and localization heads. Together, these three modules form a tightly coupled “edge enhancement–dependency calibration–discrepancy-aware fusion” pipeline, realizing the proposed edge–discrepancy collaborative design rather than a simple stacking of generic attention or multi-scale fusion blocks.

2. On the casting DR dataset, MTS-YOLOv11 achieves mAP@0.5 = 96.5% and mAP@0.5:0.95 = 68.5%, representing stable improvements of approximately 1.3 and 1.2 percentage points over the baseline YOLOv11. In multi-seed repeatability experiments, MTS-YOLOv11 maintains an mAP@0.5 level of about 96.5 ± 0.2, and its worst result still exceeds the best result of YOLOv11, indicating that the performance gain is repeatable rather than an artifact of a single run. Meanwhile, the model remains compact, with only 2.72 M parameters and 7.8 GFLOPs, and achieves an inference speed of 359.07 FPS on the same platform (versus 346.86 FPS for YOLOv11), reflecting a favorable balance among lightweight design, real-time performance, and accuracy improvement.

3. On a newly collected casting DR dataset, MTS-YOLOv11 attains mAP@0.5 = 91.3% and mAP@0.5:0.95 = 65.4%, and outperforms multiple comparative methods across all three defect types (pore, inclusion, and looseness). This demonstrates that the proposed edge–discrepancy collaborative design maintains strong cross-scenario adaptability and engineering applicability when workpiece structure, X-ray projection geometry, and background texture vary. At the same time, despite these improvements, failure case analysis shows that the model still has limitations in handling extremely dense small-target clusters and defects that severely overlap with strong structural edges. Future work may incorporate instance-level segmentation priors, stronger global context modeling, and uncertainty-aware post-processing to further enhance robustness in complex industrial inspection environments.

## Figures and Tables

**Table 1:** Comparison of Precision and Recall Rates of Different Models.

Model	Pore		Inclusion		Looseness	
	Precision/%	Recall/%	Precision/%	Recall/%	Precision/%	Recall/%
YOLOv7	71.4	81.7	62.9	68.7	77.5	76.3
YOLOv8	88.3	84.6	82.8	85.4	98.5	99.2
YOLOv11	89.3	85.6	83.9	86.4	98.7	99.5
YOLOv12	79.8	84	78.7	77.5	96.8	98.2
RT-DETR	82.2	75.4	77	73.5	88.6	91.1
MTS-YOLOv11	91.9	90.2	86.7	88.9	99.3	99.6

**Table 2:** Comparison of F1 scores for different models.

Model	F1/%		
	Pore	Inclusion	Looseness
YOLOv7	76.2	65.7	76.9
YOLOv8	86.4	84.1	98.8
YOLOv11	87.4	85.1	99.1
YOLOv12	81.8	78.1	97.5
RT-DETR	78.7	75.2	89.8
MTS-YOLOv11	91	87.8	99.5

**Table 3:** Comprehensive comparison of different models.

Model	Pore	Inclusion	Looseness	mAP@0.5/%	mAP@0.5:0.95/%	Params	FLOPs	FPS
YOLOv7	83.9	67.2	81.9	77.7	42.2	2.29M	6.6G	147.69
YOLOv8	93.3	90.7	99.2	94.4	65.7	3.01M	8.1G	264.84
YOLOv11	94.3	91.8	99.5	95.2	67.3	2.58M	6.3G	346.86
YOLOv12	90.1	85.6	99.3	91.6	59.9	2.51M	5.8G	198.54
RT-DETR	77.6	79	95.5	84.1	52.1	3.20M	8.5G	256.68
MTS-YOLOv11	95.8	94.1	99.5	96.5	68.5	2.72M	7.8G	359.07

**Table 4:** Comparison of ablation experiments in various categories.

Group	MSEES	SDAGFusion	TripletAttention	Pore	Inclusion	Looseness
0				94.3	91.8	99.5
1	√			95.6	93.6	99.5
2		√		95.2	92.7	99.5
3			√	95	92.1	99.5
4	√	√		95.5	94	99.5
5	√		√	94.9	93.2	99.5
6		√	√	95.4	92.5	99.5
7	√	√	√	95.8	94.1	99.5

**Table 5:** Comparison of ablation experiments.

Group	MSEES	SDAGFusion	TripletAttention	mAP@0.5/%	mAP@0.5:0.95/%
0				95.2	67.3
1	√			96.2	67.6
2		√		95.8	67.7
3			√	95.5	67.3
4	√	√		96.4	68.3
5	√		√	95.9	67.9
6		√	√	95.8	67.7
7	√	√	√	96.5	68.5

**Table 6:** Comparison of MSEES with simple edge baselines.

Group	Pore	Inclusion	Looseness	mAP@0.5	Params	FLOPs	FPS
0	94.3	91.8	99.5	95.2	2.58M	6.3G	346.86
1	95.6	93.6	99.5	96.2	2.57M	6.6G	243.69
2	93.7	91.9	99.4	95	3.74M	7.9G	201.19
3	94.7	93	99.5	95.7	2.43M	6.9G	159.23

**Table 7:** Repeatability Verification Results.

Model	Pore	Inclusion	Looseness	mAP@0.5	mAP@0.5(Mean ± Std)
YOLOv11	(93.81–94.27)	(91.72–92.99)	(99.40–99.50)	(95.06–95.40)	(95.2 ± 0.2)
MTS-YOLOv11	(95.83–96.27)	(93.55–95.62)	(98.38–99.50)	(96.30–96.76)	(96.5 ± 0.2)

**Table 8:** Comparison of Results from Different Models in Generalization Experiments.

Model	Pore	Inclusion	Looseness	mAP@0.5/%	mAP@0.5:0.95/%
YOLOv7	83.8	72	85.5	80.4	48.1
YOLOv8	88.2	82.7	98.1	89.7	61
YOLOv11	89.1	83.2	97.3	89.9	59.9
YOLOv12	85.1	77.5	97.6	86.7	57
RT-DETR	77.4	71.7	94	81	51.5
MTS-YOLOv11	92.3	84.1	97.6	91.3	65.4

## Data Availability

The original contributions presented in this study are included in the article. Further inquiries can be directed to the corresponding author.

## References

[1] Wang P., Jing P. (2024). Deep learning-based methods for detecting defects in cast iron parts and surfaces. IET Image Process..

[2] Hai C., Wu Y., Zhang H., Meng F., Tan D., Yang M. (2024). Approach for automatic defect detection in aluminum casting x-ray images using deep learning and gain-adaptive multi-scale retinex. J. Nondestruct. Eval..

[3] Wang D., Xiao H., Huang S. (2023). Automatic defect recognition and localization for aeroengine turbine blades based on deep learning. Aerospace.

[4] Lou L., Lu K., Xue J. (2024). Defect detection based on improved YOLOx for ultrasonic images. Sens. Imaging.

[5] Ma G., Jia H.D., Lu C.Y., Chen L.X., Zhang G.Z., Zhang L.P., Yang C. (2019). Application of Magnetic Particle Detection and Penetration Detection in Nondestructive Testing of Construction Machinery Structural Component. Nondestruct. Test..

[6] He D., Yuan J., Tang D., Wu D., Xie F., Zhang P., Yu Z. (2025). Defect intelligent detection for pipeline girth welds based on improved YOLOv5 model. Nondestruct. Test. Eval..

[7] Li L., Gao M., Tian X., Wang C., Yu J. (2024). Surface defect detection method of aluminium alloy castings based on data enhancement and CRT-DETR. IET Image Process..

[8] Pan H., Zhao H., Wei X., Zhang D., Dong B., Lan J. (2024). Study on defect detection of metal castings based on supervised enhancement and attention distillation. Mach. Vis. Appl..

[9] Pu Q.C., Zhang H., Xu X.R., Zhang L., Gao J., Rodić A., Wang Z.X. (2024). Casting-DETR: An end-to-end network for casting surface defect detection. Int. J. Met..

[10] Sainath T.N., Kingsbury B., Mohamed A.R., Dahl G.E., Saon G., Soltau H., Ramabhadran B. (2013). Improvements to deep convolutional neural networks for LVCSR. 2013 IEEE Workshop on Automatic Speech Recognition and Understanding, Olomouc, Czech Republic, 8–12 December 2013.

[11] Xue L., Hei J., Wang Y., Li Q., Lu Y., Liu W. (2022). A high efficiency deep learning method for the x-ray image defect detection of casting parts. Meas. Sci. Technol..

[12] Girshick R., Donahue J., Darrell T., Malik J. (2014). Rich feature hierarchies for accurate object detection and semantic segmentation. Proceedings of the IEEE Conference on Computer Vision and Pattern Recognition, Columbus, OH, USA, 23-28 June 2014.

[13] Ren S., He K., Girshick R., Sun J. (2015). Faster r-cnn: Towards real-time object detection with region proposal networks. Advances in Neural Information Processing Systems.

[14] Redmon J., Divvala S., Girshick R., Farhadi A. (2016). You only look once: Unified, real-time object detection. Proceedings of the IEEE Conference on Computer Vision and Pattern Recognition, Las Vegas, NV, USA, 27-30 June 2016.

[15] Yang H., Fang Y., Liu L., Ju H., Kang K. (2023). Improved YOLOv5 based on feature fusion and attention mechanism and its application in continuous casting slab detection. IEEE Trans. Instrum. Meas..

[16] Wu K., Sun S., Sun Y., Wang C., Wei Y. (2025). RBS-YOLO: A Lightweight YOLOv5-Based Surface Defect Detection Model for Castings. IET Image Process..

[17] Wang C.Y., Bochkovskiy A., Liao H.Y.M. (2023). YOLOv7: Trainable bag-of-freebies sets new state-of-the-art for real-time object detectors. Proceedings of the IEEE/CVF Conference on Computer Vision and Pattern Recognition, Vancouver, BC, Canada, 18-22 June 2023.

[18] Cui W., Li Z., Duanmu A., Xue S., Guo Y., Ni C., Zhang Y. (2024). CCG-YOLOv7: A wood defect detection model for small targets using improved YOLOv7. IEEE Access.

[19] Varghese R., Sambath M. (2024). Yolov8: A novel object detection algorithm with enhanced performance and robustness. 2024 International Conference on Advances in Data Engineering and Intelligent Computing Systems (ADICS), Chennai, India, 18–19 April 2024.

[20] Liu L., Du D., Sun Y., Li Y. (2025). SFMW-YOLO: A lightweight metal casting surface defect detection method based on modified YOLOv8s. Expert Syst. Appl..

[21] Khanam R., Hussain M. (2024). Yolov11: An overview of the key architectural enhancements. arXiv.

[22] Li X., Fan Z., Liu Q., Wan X. (2025). DSP-YOLO: An improved YOLO11-based method for steel surface defect detection. Meas. Sci. Technol..

[23] Zhang Y., Guo R., Li M. (2025). Backend-free multi-scale feature fusion network for defect detection in printed circuit board images. J. Real-Time Image Process..

[24] Ling L., Xu S., Wei L., Wei G., Jia J., Hong B. (2025). Fsd-detr: Casting surface defect detection based on improved RT-DETR. J. Real-Time Image Process..

[25] Zhao Y., Lv W., Xu S., Wei J., Wang G., Dang Q., Chen J. (2024). Detrs beat yolos on real-time object detection. Proceedings of the IEEE/CVF Conference on Computer Vision and Pattern Recognition, Seattle, WA, USA, 17-21 June 2024.

[26] Carion N., Massa F., Synnaeve G., Usunier N., Kirillov A., Zagoruyko S. (2020). End-to-end object detection with transformers. European Conference on Computer Vision, Virtual, 23–28 August 2020.

[27] Andriosopoulou G., Mastakouris A., Masouros D., Benardos P., Vosniakos G.C., Soudris D. (2023). Defect recognition in high-pressure die-casting parts using neural networks and transfer learning. Metals.

[28] Cheng S., Yang H.G., Xu X.Q., Li M., Chen Y. (2022). Improved lightweight X-ray aluminum alloy weld defects detection algorithm based on YOLOv5. Chin. J. Lasers.

[29] Wu L., Chu Y.K., Yang H.G. (2024). Aluminum alloy weld D R image defect detection technology based on YOLOv7TS. Chin. J. Lasers.

[30] Chu Y.K., Wu L., Yang H.G., Chen Y. (2025). Small Target Defect Detection in Casting DR Images Based on YOLOv8. Spec. Cast. Nonferrous Alloys.

[31] Wang Y., Yin T., Chen X., Hauwa A.S., Deng B., Zhu Y., Zhao H. (2024). A steel defect detection method based on edge feature extraction via the Sobel operator. Sci. Rep..

[32] Paris S., Hasinoff S.W., Kautz J. (2011). Local Laplacian filters: Edge-aware image processing with a Laplacian pyramid. ACM Trans. Graph..

